# Betulin-Based Oleogel to Improve Wound Healing in Dystrophic Epidermolysis Bullosa: A Prospective Controlled Proof-of-Concept Study

**DOI:** 10.1155/2017/5068969

**Published:** 2017-05-22

**Authors:** Agnes Schwieger-Briel, Dimitra Kiritsi, Christoph Schempp, Cristina Has, Hauke Schumann

**Affiliations:** ^1^Epidermolysis Bullosa Centre, Department of Dermatology, Medical Center, University of Freiburg, Freiburg, Germany; ^2^Department of Pediatric Dermatology, University Children's Hospital Zurich, Zurich, Switzerland; ^3^Research Centre Skinitial, Department of Dermatology, Medical Center, University of Freiburg, Freiburg, Germany

## Abstract

**Introduction:**

Skin fragility and recurrent wounds are hallmarks of hereditary epidermolysis bullosa (EB). Treatment options to accelerate wound healing are urgently needed. Oleogel-S10 contains a betulin-rich triterpene extract from birch bark. In this study, we tested the wound healing properties of topical Oleogel-S10 in patients with dystrophic EB.

**Methods:**

We conducted an open, blindly evaluated, controlled, prospective phase II pilot trial in patients with dystrophic EB (EudraCT number 2010-019945-24). Healing of wounds treated with and without topical Oleogel-S10 was compared. Primary efficacy variable was faster reepithelialization as determined by 2 blinded experts. The main secondary outcome variable of the study was percentage of wound epithelialization.

**Results:**

Twelve wound pairs of 10 patients with dystrophic EB were evaluated. In 5 of 12 cases, both blinded reviewers considered epithelialization of the intervention wounds as superior. In 3 cases, only one reviewer considered Oleogel-S10 as superior and the other one as equal to control. Measurements of wound size showed a trend towards accelerated wound healing with the intervention but without reaching statistical significance.

**Conclusion:**

Our results indicate a potential for faster reepithelialization of wounds in patients with dystrophic EB when treated with Oleogel-S10 but larger studies are needed to confirm significance.

## 1. Introduction

Epidermolysis bullosa (EB) comprises a heterogeneous group of inherited skin diseases characterized by skin fragility leading to recurrent wounds [1, 2]. Cutaneous and extracutaneous manifestations can result in severe morbidity and a reduced life expectancy [3–6]. Particularly in the generalized dystrophic EB subtypes, there is a significant risk of developing aggressive squamous cell carcinomas, with an increased incidence of metastases and death [7].

Up to now, no causal therapy in the sense of prevention of blister formation or improvement of skin stability is available. Treatment of EB mostly remains symptomatic [8] with optimal wound care and protection of the skin being the core therapeutic strategies. Wound care in these patients usually follows the “wound bed preparation model” [9–11], including the whole patient centered concerns [12], as well as local wound factors such as reduction of bacterial load and choice of optimal atraumatic dressings. Additional means to accelerate wound healing are urgently needed [13].

Birch (*Betula* species) is a medical plant. Its bark has been used as a natural remedy for skin diseases and wound care for centuries [14, 15]. Oleogel-S10 is a semisolid gel, containing 10% triterpene dry extract (TE) from* Betulae* cortex (birch bark) and refined sunflower oil (SFO) without the need for further excipients [16].

Both components have a low potential for allergic sensitization and seem therefore suitable for use on wounds. To our knowledge, over the course of 10 years, there have been 2 cases of contact sensitization towards the triterpene extract, one of them being published [17]. Both patients known to us showed only a local skin reaction. No anaphylaxis has been reported.

Refined SFO is also considered to have a very low potential for sensitization and is therefore often used as a moisturizer in little children [18]. In an older study by Halsey et al., SFO did not provoke a reaction when tested in sunflower seed allergic patients [19].

Triterpenes were shown to enhance epidermal barrier recovery and to stimulate wound healing [20–24]. In 2014, Ebeling et al. published results around the molecular mechanism of the effects of birch bark on keratinocytes [25]. The authors showed significantly accelerated reepithelialization in a porcine ex vivo wound healing model when treating the wound with Oleogel-S10 as compared to sunflower oil alone or the oil in combination with a gelling agent (ethyl cellulose). In the same model, this treatment led to an improvement of barrier regeneration. Various mediators involved in the inflammatory phase of wound healing were positively modulated, among them COX-2 and IL-6, the latter being known for playing an important role in wound healing and epidermal barrier repair [26, 27]. In addition, in vitro and in vivo studies suggest that betulin has anticarcinogenic properties and induces apoptosis in different tumor cells including human squamous cell carcinoma (SCC) cells [28–31].

The ideal formulation of Oleogel-S10 has further been examined by Steinbrenner et al. [32]. The group tested the effect of different oils on wound healing when used alone or in combination with TE. The majority of oils seemed to hinder wound healing to some extent, SFO being among the least impairing oils when used alone. However, when using SFO with TE as in Oleogel-S10, wound healing was significantly improved as compared to both sunflower oil (SFO) and SFO with ethyl cellulose for an improved viscosity.

The effects of Oleogel-S10 have already been investigated in vivo on different types of wounds where this treatment seemed to promote wound healing and was very well tolerated [33–35]. Additionally, a very recent randomized controlled trial found enhanced epithelialization of split-thickness skin graft donor site wounds after treatment with Oleogel-S10 [22]. In 2016, Oleogel-S10 was approved in the European Union as a new medicine for the treatment of partial thickness wounds in adults (http://www.ema.europa.eu/ema/index.jsp?curl=pages/medicines/human/medicines/003938/human_med_001956.jsp).

**Figure 1:**
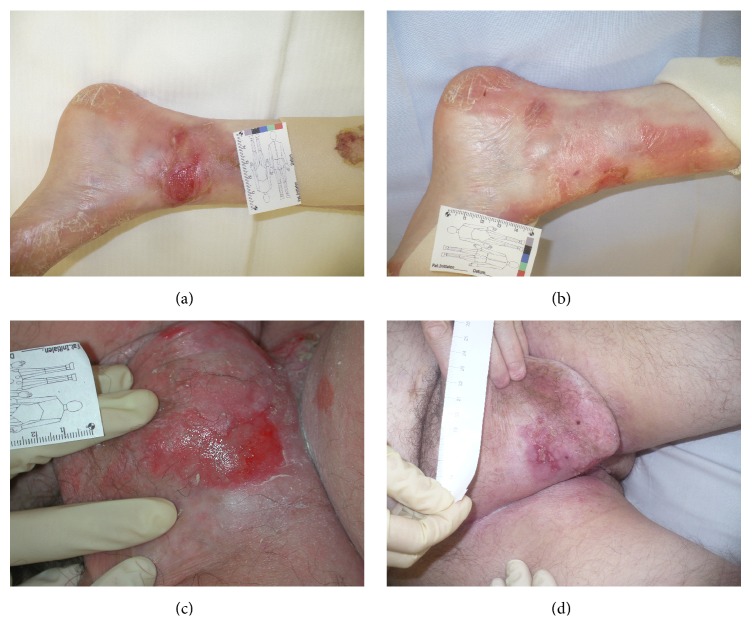
Healing of chronic wound on the ankle of a 12-year-old suffering from RDEB after 5 weeks of treatment with Oleogel-S10 (a-b). Healing of chronic wound in the groin of a 50-year-old male after only 6 days of treatment with Oleogel-S10 (c-d).

The results of these different reports were in keeping with our own experiences from using Oleogel-S10 for wound care of few individual patients suffering from different subtypes of EB outside of a study setting (Figure 1).

In the present study, we investigated the effect of Oleogel-S10 on wound healing in patients with dystrophic EB. We hypothesized that Oleogel-S10 in combination with standard nonadhesive wound dressings would lead to faster wound healing as opposed to the use of standard wound care alone. Furthermore, we assessed the feasibility of conducting a larger trial in this patient group.

## 2. Methods

**Figure 2:**
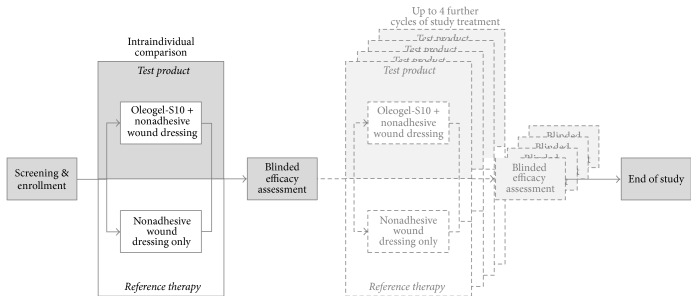
Study design.

This study was designed as an open-label, prospective, controlled, blindly evaluated, monocentric phase II pilot trial to compare intraindividually the efficacy and tolerance of Oleogel-S10 in combination with nonadhesive wound dressing versus nonadhesive wound dressing alone (Figure 2). The intraindividual comparison was chosen to minimize the influence of age, health status, medications, and other potentially confounding factors.

The study (EudraCT number 2010-019945-24) was conducted in compliance with IEC, informed consent regulations, and ICH and GCP guidelines and was approved by the ethics committee of the University of Freiburg.

### 2.1. Patient Eligibility Criteria

Patients of any age beyond infancy and with an immunochemically and/or genetically proven diagnosis of hereditary EB and at least 1 wound between 10 cm^2^ and 200 cm^2^ (alternatively 2 comparable lesions of at least 5 cm^2^ each) were recruited for this study. We differentiated wounds that had appeared less than 6 weeks earlier and those with no tendency to heal for at least 6 weeks. Children were deliberately included in this study, as they represent a large proportion of our patients and optimized wound healing would be of particular interest to them.

Patients were excluded from this study if they had been on a systemic treatment with corticosteroids within the last 30 days or if they had taken any investigational drugs within 3 months before screening. They were also excluded if they suffered from uncontrolled diabetes mellitus, diabetic ulcers, or other diseases or conditions that could interfere with the assessment of safety, tolerance, or efficacy of Oleogel-S10 during the study.

Patients or their legal representatives had to provide written informed consent before participation in the study. For this proof-of-concept study, we aimed to include 10 patients.

### 2.2. Intervention

Patients of all ages presenting at the EB-Centre Freiburg for regular follow-up visits were screened according to the study protocol. If the investigator identified a skin lesion (10 cm^2^ to 200 cm^2^) eligible for study treatment, it was divided into two halves which were then allocated to either the intervention or the treatment arm. This method ensured a comparison between wound areas in a similar anatomic location and within identical wound healing phases. Alternatively, if there were two comparable lesions (≥5 cm^2^ each) of similar size and shape, these were selected and considered one wound pair. Wounds located at sites of major trauma (e.g., elbows, knees, and buttocks) and circumferential wounds were not included.

One half of the wound was chosen at random to be treated with Oleogel-S10 (applied approximately at 1 mm thickness) and covered by a nonadhesive wound dressing (Mepilex Transfer®, Mölnlycke Health Care, Sweden); the unwounded skin next to this half as well as the border between the wound halves was marked to ensure that Oleogel-S10 was always applied to the same part of the wound. In other prior studies, it had been shown that the product did not spread to other areas than the ones it was applied to. Each wound half was measured and the data were entered into a wound surface-measuring program developed at our wound care clinic (University of Freiburg, Department of Dermatology) to ensure a comparable initial wound size. To serve as control, the other half of the lesion was covered with a nonadhesive wound dressing only (Mepilex Transfer, reference therapy). If the investigator had identified two comparable wounds, these were treated correspondingly. The intervention and control treatment were applied in an open-label fashion.

Dressings were changed based on the study flow chart every 24 to 48 hours until the end of treatment at day 14. In case of delayed wound healing (wound present > 6 weeks at initiation of study treatment), the treatment period was prolonged until day 28.

### 2.3. Outcome Assessment

Before the start of treatment and at every dressing change, photographs of the respective wounds were taken. We used a Canon camera, EOS Digital SLR series, and our in-house wound measuring system, which allowed outer tracing of wound borders and calculation of wound surface.

The images were uploaded into the electronic Case Report Form (eCRF), cropped to remove pen markings at the treatment side, and coded for blinding. The (open) wound surface area (in cm^2^) was recorded on day 0 and at each dressing change until the end of the treatment period at day 14/day 28. These data enabled us to calculate the percentage of reepithelialization. Two independent experts evaluated treatment efficacy in a blinded fashion based on comparing chronological series of cropped wound photographs taken before the start of treatment, during wound dressing changes, and at the end of treatment on day 14/day 28 and choosing the series with signs of better reepithelialization. Remote photographic analysis has been shown to be a reliable method of wound evaluation [36].

The degree of reepithelialization was calculated as the ratio of reepithelialized wound area relative to the initial wound area.

At each dressing change, severity of touch sensitivity and itch of each treatment area were examined and rated on a visual analog scale from 1 to 10. Accordingly, patients or their legal representatives were asked to provide feedback on tolerability and efficacy of the intervention and control treatment. The amount of exudate was rated as low, middle, or strong.

### 2.4. Primary Outcome

All wound photographs were summarized within chronological series of even orientation and size by patient and wound. The 2 blinded experts were asked to evaluate reepithelialization of the wound pairs for each series and to decide which part of the wound reepithelialized faster than the other or if there was an equal degree of reepithelialization in both parts of the wound pair.

### 2.5. Secondary Outcomes

Secondary efficacy variables of the study were percentage of wound epithelialization as measured and documented at each dressing change. The degree of reepithelialization was calculated for each wound as the ratio of reepithelialized area to initial open wound area. Wounds were considered closed if they were at least 95% epithelialized.

Additionally, we evaluated the extent of touch sensitivity, itch, exudation, and the assessment of efficacy and tolerance given by the investigators and patients or their legal representatives.

Secondary safety variables of the study were all adverse events, treatment-related or not, graded according to the NCI-CTC grading system on a 4-point scale (mild, moderate, severe, and life-threatening) and assessment of tolerability by the investigators and patients and/or their legal representatives.

### 2.6. Statistical Considerations

A sample size calculation was not performed, as the planned number of patients eligible for enrollment into this pilot study was limited. Statistical analyses on the primary outcome parameter of faster reepithelialization were performed using a two-sided exact binomial test with a significance level of *p* = 0.05 against the null hypothesis of no difference between treatments. For the secondary outcome variable of the amount of reepithelialization, we applied Wilcoxon signed-rank test.

## 3. Results

### 3.1. Patient Data

**Figure 3:**
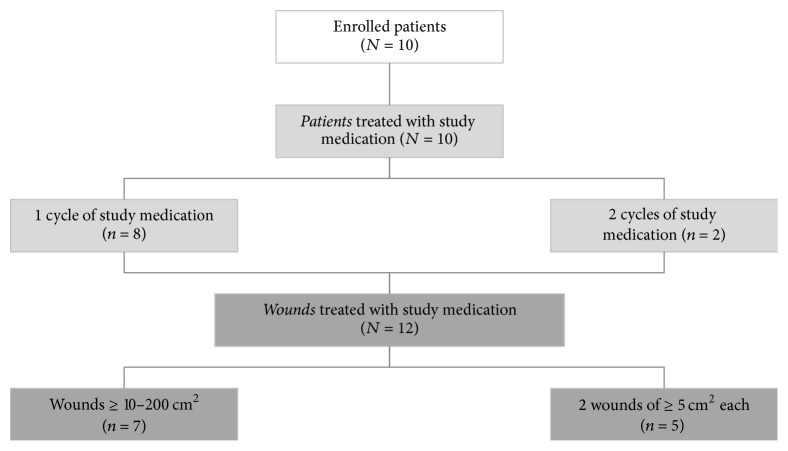
Disposition of patients.

10 patients were enrolled in the study. As 2 patients participated in a second cycle of treatment, 12 wound pairs were evaluated. None of the patients was lost to follow-up or dropped out (Figure 3): all patients who qualified for the inclusion criteria suffered from dystrophic EB, 9 patients had recessive dystrophic EB (RDEB), one had intermediate generalized RDEB, 8 had severe generalized RDEB, and 1 had localized dominant dystrophic EB. Expression of collagen VII and/or mutations of* COL7A1* were determined as summarized in Table 1. The median age of patients was 20 years (range: 6–48 years). No infant screened during the study period showed large enough wounds enabling participation.

### 3.2. Wounds

Nine of 12 wound pairs (75.0%) analyzed in the study were present in less than 6 weeks. 3 of 12 wound pairs (25%) were chronic, showing no tendency to heal for more than 6 weeks. The mean wound size was 17.5 cm^2^ in the intervention group (7.3–45 cm2) versus 17.7 cm^2^ in the control group (6.3–45 cm^2^). In 7 cases, large wounds were chosen and divided into two halves for comparative treatment. In the other 5 cases, 2 comparable wounds were identified, where one was treated with Oleogel-S10 and the other with nonadhesive wound dressing alone (Table 2). All patients followed the study protocol except for one patient, whose wound was additionally treated with an antiseptic gel (polyhexanide 0.04%, Lavasept®) on* both* areas for the duration of 3 dressing changes towards the end of the study due to a suspected superinfection.

### 3.3. Reepithelialization

In 5 of 12 cases (41%), the intervention wound was rated as better epithelialized unanimously by both independent reviewers. In another 3 of 12 cases, 1 reviewer rated epithelialization of the intervention wound as superior whereas the other expert considered the wounds as healing equally well. In the remaining 4 cases, either epithelialization was considered as equal or the result was controversial (Table 3). Thus, the comparison of unanimous “winners” was 5 versus 0 (*p* = 0.063, binomial test) in favor of Oleogel-S10 compared to standard-of-care treated wound halves.

### 3.4. Secondary Outcomes

**Figure 4:**
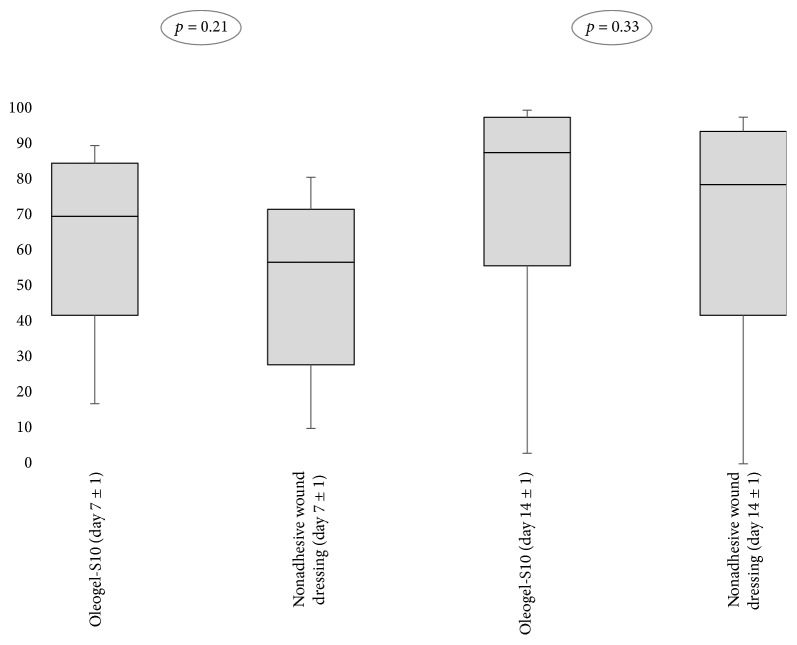
Percentage of wound area with a newly formed epithelial layer on day 7 ± 1 and day 14 ± 1.

Measurement of wound sizes suggested a trend towards faster closure of wounds that had been treated with Oleogel-S10 both on day 7 and on day 14. On day 7 (±1), the median epithelialization of the initial wound surface was 69.7% (intervention) versus 57.4% (control) and on day 14 was 87.7% (intervention) versus 79.2% (control) (*p* = 0.21 on day 7; *p* = 0.33 on day 14, Wilcoxon test, Figure 4).

**Figure 5:**
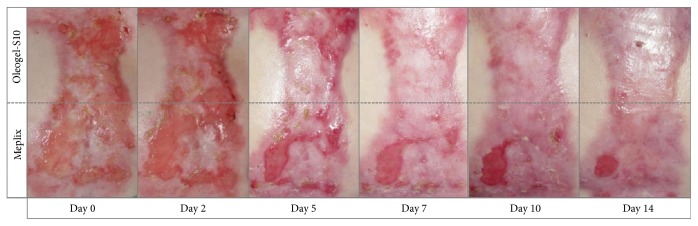
Complete wound closure of the intervention wound at day 13 of treatment.

Wound closure with at least 95% epithelialization was reached in 5 of 12 wounds in the Oleogel-S10 group versus 2 of 12 in the control group. The mean time for wound closure was 10.5 days in the intervention group versus 14 days in the control group (Figure 5).

As can be expected in EB patients, we experienced retraumatization of wounds with an increase of wound size ≥5% compared with the preceding measurement in 7 of 12 wound pairs. In 3 wound pairs, it occurred on both sides, the intervention and the control wound. Additionally, it occurred in another 3 intervention and 1 control wound halves. As this made the evaluation of reepithelialization more difficult, we looked at the epithelialization of all wounds at one follow-up prior to retrauma and compared the mean percentage of epithelialization at this point, which was 78% for Oleogel-S10 versus 73% for the control. There was an advance of epithelialization by ≥10% in 5 of 12 cases for Oleogel-S10 as opposed to 2 of 12 cases for the control.

Patients' assessment of tolerability of Oleogel-S10 and standard treatment was considered as good (97.4%) or acceptable (2.6%) in the intervention and as good in 100% of the control group. Patients reported treatment with Oleogel-S10 more frequently as efficient (75.3%) than with standard dressings alone (53.3%). The application of Oleogel-S10 was easy. The substance was tolerated very well without any complaints of stinging or burning.

We did not find any relevant differences in the perception of touch sensitivity, itch, and amount of exudate between intervention and control arms.

## 4. Discussion

In this phase II pilot study, we investigated the effects of Oleogel-S10 on wounds of patients suffering from dystrophic EB. Both the evaluation of two blinded experts and the percentage of epithelialization measured by the investigators were consistent and suggested a trend towards faster healing of wounds when treated with Oleogel-S10 combined with nonadhesive dressings versus standard wound care with the dressings alone. The number of healed wounds (≥95% epithelialization) was higher and the mean time needed for wound closure was shorter in the intervention than in the control group. Likely owing to the relatively low number of 10 patients, however, these results did not reach statistical significance.

The results of this pilot trial showed feasibility of study procedures and call for larger randomized controlled trials to confirm the promising effects of Oleogel-S10 application in EB wounds.

Recruitment of patients was achieved within the scheduled period and patients were compliant, cooperative, and motivated to fulfil this study. Also, the duration of the study between 2 and 4 weeks was feasible for all patients. None of them dropped out or was lost to follow-up, which proved the good tolerability of the product and the great need for new therapeutic measures to treat wounds in patients with EB. However, healing of large wounds exceeded the anticipated 4 weeks, which led to a rather low number of fully epithelialized wounds after the completed study period of 2 and 4 weeks.

Tolerability of the study medication was very good. We did not experience any local or generalized irritability or contact sensitization towards either of the two components of Oleogel-S10. Certainly, any medically active ingredient has the potential for allergic sensitization, especially when applied onto open wounds. Therefore, in patients with known sunflower seed allergy, Oleogel-S10 should be used with caution.

According to the study protocol, we included the first 10 patients willing to participate in the study. As the majority of patients who present at our center suffer from dystrophic EB, we included only this EB subtype in our study. This allowed a more homogenous study population sharing characteristic traits of this rare disorder. However, it is possible that our results are not applicable to other EB types.

Dystrophic EB causes major morbidity for the affected individuals leading to multiple secondary complications such as anemia and deficiencies of iron, zinc, vitamins, and micronutrients that impair wound healing. Therefore, the intraindividual control, comparing wounds or wound halves of the same individual, was a suitable approach, eliminating confounding factors.

When planning the study, we chose to compare Oleogel-S10 to standard treatment instead of using the vehicle, respectively, placebo, as control. Apart from betulin extract, the only other ingredient of Oleogel-S10 is sunflower oil (SFO) which, other than Oleogel-S10, does not stay in the allocated wound half due to its liquid consistency. Also, in recent studies, a significantly better effect on wound healing of Oleogel-S10 as opposed to both SFO and SFO with ethyl cellulose has been demonstrated [32]. The use of pure SFO has, as have the large majority of pure oils, even shown a mild negative effect on wound healing as opposed to control making pure SFO a less promising candidate as control than standard wound care.

## 5. Conclusion

Oleogel-S10 is a topical gel made of pure sunflower oil and a 10% triterpene extract from birch bark. We used Oleogel-S10 on acute and chronic wounds (>6 weeks) in 10 patients with dystrophic EB.

The results of the present study were promising and provided reason for further investigations of the effects of Oleogel-S10. Larger studies using Oleogel-S10 for wounds of patients with all different forms of EB are needed to clarify the effectiveness and safety in EB in general.

Concurrent to additional clinical studies with Oleogel-S10, further insights into the anticarcinogenic effects of betulin on human SCC cells, ideally coming from EB patients, are needed. The anticarcinogenic effect, if proven to be real, in combination with wound healing effects would make Oleogel-S10 an ideal substance to use in EB wounds at early stages, especially in severe generalized RDEB, the subtype with the highest number of aggressive and metastasizing SCCs.

The above-mentioned additional studies would help to identify the optimal indications for use of Oleogel-S10 and to clarify whether it can stand up to its promises.

## Figures and Tables

**Table 1:** Patient data.

Number	Age (years)	Gender	Type of EB	Collagen VII staining (IFM)	Mutation cDNA	Mutation protein	Skin involvement
1	6	M	RDEB (SG)	Negative	c.3141delG homozygous	p.Cys1048 Alafs^*∗*^61	Generalized blistering, scarring, mutilations
2	10	F	RDEB (SG)	Not done	c.4590delA homozygous	p.Gly1531 Glufs^*∗*^179	Generalized blistering, scarring, mutilations
3	16	F	RDEB (SG)	Not done	c.4590delA homozygous	p.Gly1531 Glufs^*∗*^179	Generalized blistering, scarring, mutilations
4	9	M	RDEB (SG)	Not done	c.1934delC homozygous	p.Pro645 Glnfs^*∗*^45	Generalized blistering, scarring, ++pseudo synechiae
5	36	M	RDEB (SG)	Negative	425A>G homozygous	p.? Altered splicing	Generalized blistering, scarring, mutilations
6	47	F	RDEB (GI)	Reduced	c.[3832-2A>G]; [4039G>T]	p.[?]; [G1347W]	Generalized blistering, scarring, mild proximal pseudo synechiae
7	21	M	DDEB (loc.)	Normal	c.7868G>T	p.Gly2623Val	Blistering on lower legs, nail dystrophy
8	28	M	RDEB (SG)	Not done	Not done	Not done	Generalized blistering, scarring, mutilations
9	20	M	RDEB (SG)	Not done	c.[2212_2215dup]; [7621C>T]	p.Glu739 GlyfsX2	Generalized blistering, scarring, mutilations
10	17	M	RDEB (SG)	Negative	c.[425A>G]; [1837C>T]	p.[?]; [Arg613X]	Generalized blistering, scarring, mutilations

EB: epidermolysis bullosa; DDEB: dominant dystrophic EB; RDEB: recessive dystrophic EB; SG: severe generalized; GI: generalized intermediate; loc.: localized; IFM: immunofluorescence mapping.

**Table 2:** Wound characteristics.

Patient data				Wound characteristics			
Number	Age (years)	Gender	Type of EB	Wound number	Type	Location	Age of wound
1	6	M	RDEB (SG)	1	Large divided	Lower extremity	Recent
2	10	F	RDEB (SG)	1	Large divided	Lower extremity	Recent
3	16	F	RDEB (SG)	1	Large divided	Upper extremity	Recent
4	9	M	RDEB (SG)	1	Large divided	Lower extremity	Chronic
5	36	M	RDEB (SG)	1	Large divided	Upper extremity	Recent
6	47	F	RDEB (GI)	1	Separate	Trunk	Chronic
7	21	M	DDEB (loc.)	1	Large divided	Lower extremity	Recent
8	28	M	RDEB (SG)	1	Separate	Trunk	Chronic
9	20	M	RDEB (SG)	1	Separate	Trunk	Recent
9	20	M	RDEB (SG)	2	Separate	Trunk	Recent
10	17	M	RDEB (SG)	1	Large divided	Trunk	Recent
10	17	M	RDEB (SG)	2	Large divided	Lower extremity	Recent

EB: epidermolysis bullosa; DDEB: dominant dystrophic EB; RDEB: recessive dystrophic EB; SG: severe generalized; GI: generalized intermediate; loc.: localized; recent: less than 6 weeks; chronic: longer than 6 weeks.

**Table 3:** Results of blinded efficacy evaluation by patient and wound.

Number	Reviewer 1	Reviewer 2	Unanimous “winner”	
			Oleogel-S10	Wound dressing
1	Control	Oleogel-S10	—	—
2	Oleogel-S10	Equal	—	—
3	Oleogel-S10	Oleogel-S10	1	—
4	Oleogel-S10	Equal	—	—
5	Equal	Equal	—	—
6	Oleogel-S10	Oleogel-S10	1	—
7	Equal	Equal	—	—
8	Oleogel-S10	Control	—	—
9 (1)	Oleogel-S10	Oleogel-S10	1	—
9 (2)	Equal	Oleogel-S10	—	—
10 (1)	Oleogel-S10	Oleogel-S10	1	—
10 (2)	Oleogel-S10	Oleogel-S10	1	—

*Control* = nonadhesive wound dressing.

## Abbreviations

EB: Epidermolysis bullosa
RDEB: Recessive dystrophic EB
DDEB: Dominant dystrophic EB
SG: Severe generalized
GI: Generalized intermediate
TE: Triterpene extract.
